# Whither compassionate leadership? A systematic review

**DOI:** 10.1007/s11301-023-00340-w

**Published:** 2023-04-04

**Authors:** Sunder Ramachandran, Sreejith Balasubramanian, Wayne Fabian James, Turki Al Masaeid

**Affiliations:** 1 Abu Dhabi School of Management, Abu Dhabi, UAE; 2 Middlesex University, Dubai Campus, Dubai, UAE

**Keywords:** Compassionate leadership, Compassion, Leadership, Systematic review, Leadership styles, D02, D23, J24, J28, L21, L25, M12, M54

## Abstract

Recent disastrous events, such as the COVID-19 pandemic, has amplified the appeal for compassionate leadership in organizations and is viewed as the need of the hour. While compassion is a timeless concept, there appears to be a lack of clarity around the idea of compassionate leadership, and the current understanding appears limited, fragmented, and scattered across the literature. This integrative systematic review of 41 articles during the last 20 years (2002–2021) conceptualizes six critical dimensions of compassionate leadership, namely: empathy; openness and communication; physical, mental health and well-being; inclusiveness; integrity; respect and dignity. These findings enhance our understanding of how organizational leaders can effectively incorporate the dimensions to become compassionate leaders. The review examines how compassionate leadership is defined and understood in the literature, and a contribution in the form of a definition for compassionate leadership is offered. Finally, gaps in the literature are highlighted, offering avenues for future research, along with implications for research, theory, and practice.

## Introduction

Leadership has been one of the most comprehensively studied scientific disciplines for over a century, transcending cultures, domains and dimensions through social, psychological and organizational perspectives (Hogg 2001; King 1990). Indeed, there are many hues of leadership which have been examined and like a colourful tapestry they baffle more, than clarify the mystique of leadership. In an attempt to explain the complex nature of leadership a wide variety of theoretical approaches have been studied. Scholarly studies have conceptualized leadership from several viewpoints. Some considered it from the perspective of a process, a trait, a behaviour while others from an information-processing and relationship perspective. Among many leadership styles, one of the styles that has started to receive considerable attention in the business world-and duly amplified by the calamitous impact of COVID-19- is compassionate leadership. In a world beset with uncertain times where the future of our jobs and uncertain economic conditions are giving rise to anxiety, stress, insecurity, burnout levels to an unprecedented level compassionate leadership has been mooted as the panacea to address the suffering, pain and toxicity being perpetuated and experienced by the workforce at their workplaces (Frost 2003; Gallo 2020; Moss 2021; Rao and Sutton 2020; Trapp 2019). Compassionate leadership is viewed as a leadership that embodies an act of love, care, and selflessness and enables a caring and supportive organization (Oruh et al. 2021). A compassionate leader has a genuine interest in seeing their people not just perform but thrive. Compassionate leadership is an interpersonal process involving the noticing, feeling, sense making, and acting by the giver in a way that alleviates the suffering of the receiver (Banker and Bhal 2018).

When employees experience compassion at their workplace in times of suffering, they receive many benefits. For instance, employees feel legitimized and elevated; recover more quickly; develop confidence in being valued by their organizations; feel more satisfied in their jobs; experience positive emotions at work; reciprocate with compassion towards others; feel connected to their organizations; generate cooperation amongst others and create a conducive environment of respect and harmony (Lilius et al. 2013). These benefits in turn lead to enhanced productivity and better organizational performance. Leadership at workplace is inexorably linked not only to the organizational productivity and performance, but also to the well-being and health of its workforce. Organizational leaders’ responsibility towards their organization and workforce gets magnified during times of crisis, such as the pandemic. It is the leader’s job to prevent a disaster from turning into a catastrophe by building cultural and psychological protections for the employees (Rao and Sutton 2020). Compassion is expected to create a sense of togetherness during the crisis. It is little wonder that during such times one sees increased cries for compassion and succour at workplaces. Hence, it is timely to explore the relevance of compassionate leadership especially during times of crisis, wherein the future of our jobs, the economies, and the world is hazy. The demonstration of empathy and walking compassionately in the shoes of employees is critical during times in which human tragedy is frequent.

Unfortunately, despite the significance of compassionate leadership for organizations, academic research is limited and fragmented. Past studies on compassionate leadership have been mostly in the field of health and medical industry demonstrating a significant gap in the business domain. Any efforts to conceptualize the critical dimensions of compassionate leadership are still in the nascent stage. These limitations make it difficult for researchers and practitioners to appreciate the progress made in the compassionate leadership literature; to derive comprehensive and objective insights; to forge a path ahead for theoretical, empirical advancement and, to develop a practitioners' guide (Balasubramanian and Fernandes 2022).

Webster and Watson (2002) believe that tackling an emerging issue could benefit from exposure to potential theoretical foundations resulting in the development of a proposed conceptual model. The significance of compassionate leadership as an emerging issue is relevant, pertinent and a better understanding is needed. A systematic review is considered as a suitable fit when one seeks evidence in the literature to answer specific research questions. It is a methodology that is more rigorous than literature reviews, is scientific, transparent, replicable and limits the subjectivity and bias of the researchers (Kuckertz and Block 2021; Pizzolitto et al. 2022; Tranfield et al.^2003^).

Several studies have systematically reviewed different leadership styles. For example, Pizzolitto et al. (2022) in their systematic review analyse fifty four articles to explore the effects of authoritarian leadership styles on performance by using a protocol developed by Wolfswinkel et al. (2011). Their analysis reveals that authoritarian leadership can negatively affect team performance; can compromise the team’s psychological self-confidence, and thereby worsen performance. Their analysis also reveals that the interest in the field in the last two decades has shifted from Western to Eastern countries as an affirmation of the prevalence of authoritarian leadership styles in most eastern businesses. Williams et al. (2022) focused on leader credibility based on an analysis of 108 articles where they find that leader credibility is not consistently defined or measured. Rudolph et al. (2020) present a critique on healthy leadership while comparing various models of ‘health leadership’. Their review of 35 articles finds methodological issues, unclear procedures for scoring measures and a lack of critical approach for introducing new concepts or discussing research findings. Frangieh and Yaacoub (2017) explore the topic of responsible leadership, its challenges, outcomes and practices while Haque et al. (2021) in their review on responsible leadership offer a conceptual framework to propose a relationship between presenteeism and employee turnover. Eva et al. (2019) analyse 270 articles to provide a conceptual clarity of servant leadership in comparison to other leadership styles. They propose a definition of servant leadership and evaluate 16 measures of servant leadership. Pearson-Goff and Herrington (2014) discuss what police leadership is, their characteristics and activities through their review of 66 articles. Bush and Glover (2016) examine literature on school leadership and management in South Africa linked to the 20th anniversary of the democratic government and integrated education. Although there are several systematic reviews on different leadership styles, we did not find any review on compassionate leadership. In summary, the significance of compassionate leadership for organizations, limited academic research in the business domain and a lack of any systematic review for this timeless and timely concept pointed us to study the extant literature for this emerging phenomenon.

Thus, this study aims to identify and resolve definitional ambiguities, provide an integrated and synthesized overview of the current state of knowledge, evaluate existing methodological approaches, describe research insights and gaps, and provide future research directions. Integrating existing knowledge into a comprehensive model allows investigation of multiple theoretical perspectives simultaneously and allows knowledge to grow consistently in the field (Webster and Watson 2002). The results will provide organizational leaders with valuable and actionable insights on compassionate leadership. This study addresses the following research questions:

1. How is compassionate leadership defined and viewed in the literature?
2. How do we measure compassionate leadership?
3. What are the critical dimensions of compassionate leadership?
4. What are the follower and organizational benefits of compassionate leadership?
5. What is the future of compassionate leadership research?

Following this introduction, the rest of the paper is organized as follows: Sect. 2 provides a brief background on compassion at the workplace; Sect. 3 explains the research methodology; Sect. 4 discusses the findings based upon the research questions and limitations of the study; and finally, in Sect. 5 we discuss the implications.

## Background on compassion at the workplace

Since Frost’s (1999) call there has been a growing body of research devoted to examining compassion at workplace. These studies have argued in favour of workplace compassion as an alternate paradigm over the traditional motivation and rationale-based management approaches (Guinot et al. 2020). Workplace compassion has been defined as the interpersonal process involving the noticing, feeling, sense-making, and acting in a way that alleviates the suffering of another person and has been mooted as a panacea to address the suffering and help develop organizational performance (Dutton et al. 2014; Guinot et al. 2020).

The earliest stream of studies comprised of essays passionately arguing in support of compassion at the workplace (Dutton et al. 2005, 2006; Frost 1999, 2004, 2006; Kanov et al. 2004; Lilius et al. 2003, 2008). Workplaces are the environment in which we live and breathe our professional lives. These environments can be sources of inspiration, learning, enjoyment and fun, or/and of pain, suffering, intrigue, conspiracies, frustration, harm or other debilitating effects (Gersick et al. 2000). Avramchuk et al. (2013) viewed compassion as being helpful in organizational change and development. Paakkanen et al. (2021) find that compassion skills could be developed through emotional training skills. Guinot et al. (2020) in their empirical study test the direct and indirect effects of compassion on organizational performance and relationships. Others have focused on compassion practices at business organizations on how managers construe the meaning of compassion at their workplace (Banker and Bhal 2018). Wei et al. (2016) develop a theoretical framework for understanding compassion and Papazoglou et al. (2019) focus on compassion satisfaction and fatigue among police officers.

Experiencing compassion at the workplace strengthens the bond between the workers psychologically; there is increased commitment to the organization, and co-workers demonstrate supportive behaviour to their colleagues. Compassion helps in reducing burnout and fatigue, improves the well-being and health of the workforce, strengthens the relationship among the employees and contributes to the overall productivity in an organization (Poorkavoos 2016). Other studies have also pointed out to improved performance, trust, commitment, customer satisfaction, positive emotions, employee engagement, prosocial behaviour’s, employee attraction and retention contributing to their competitive advantage in the long run (Guinot et al. 2020). Compassionate leaders would not only understand but proactively ask and seek information to place themselves in the shoes of their direct reports and act to lighten the suffering. Balasubramanian and Fernandes (2022) pointed out that compassion and care create a sense of togetherness during a crisis. Bavel et al. (2020) argue that leaders' ability to develop and embed a sense of "us-ness" among followers is essential during the COVID-19 pandemic. A leader's emphasis during a crisis should be on making a connection rather than correction. Moreover, the leaders must have the humility to listen to the wide range of employee concerns during a crisis. It shows that leaders genuinely care about them and their wellbeing, including mental, emotional, and physical while making decisions (Balasubramanian and Fernandes 2022). The manner in which a leader and his/her organization manages a crisis is especially influenced by the leaders tendencies to perceiving and reacting to stakeholder’s emotions (König et al. 2020).

Thus, one may opine that compassion at the workplace has an ameliorating effect on an organization’s performance. Since leadership has an important role in creating a culture of compassion at the workplace, understanding the phenomenon of compassionate leadership is worthy and timely to uncover unexamined dimensions of the concept and suggest important practical implications for practice.

## Research methodology

A systematic literature review methodology was adopted to answer the research questions. A systematic review brings together all existing research, follows rigorous processes and methods to focus on a specific question/s integrates and, interprets the findings to increase understanding (Denyer and Tranfield 2009; Fisch and Block 2018; Kuckertz and Block 2021; Tranfield et al. 2003). It is interesting to note how different studies have followed different steps in conducting a systematic review and these criteria’s baffle more than provide a generalized approach. For instance Baarspul and Wilderom (2011) and Khandker (2022) describe the identification or searching the research papers as the first step. Tranfield et al. (2003) divide the review process into three stages; Stage I- Planning the review; Stage II- Conducting the review; Stage III- Reporting and dissemination. In Tranfield et al. (2003) the identification/search of research appears in the second stage. Torgerson (2003) propose seven main stages of a systematic review—well established in the health care, social policy and education—and suggests starting with the protocol or plan of the research; literature search and screening; ‘scoping’ or ‘mapping’ the research; extracting the data; synthesis; and finally presenting the report. Wolfswinkel et al. (2011) propose a five-stage process using grounded theory as a method. We mostly adopt the stages and criteria proposed by Tranfield et al. (2003), Kuckertz and Block (2021), Fisch and Block (2018) and Wolfswinkel et al. (2011). Tranfield et al. (2003) has one of the highest citations (n = 10,573) which reveals the high influence of their work (Khandker 2022), while Kuckertz and Block (2021) and Fisch and Block (2018) acknowledge a lack of clear guidelines and provide the criteria’s for reviewing the articles in management and business research. The following section focuses on how the review was conducted.

### Conducting the review

The review panel consisted of the four authors who have extensive industry experience in the field of leadership and academia. By virtue of their senior leadership positions at various organizations, they have a solid grasp of leadership and its nuances. The initial stages of review were an iterative process wherein the concept of compassionate leadership was discussed and clarified amongst the authors and a consensus was reached on the importance of such a study to be conducted.

#### Identification of research (article extraction)

As recommended by Webster & Watson (2002) we conducted an extensive search to determine the articles for the review and are discussed below:

Identification of databases and Search scope

**Fig. 1:**
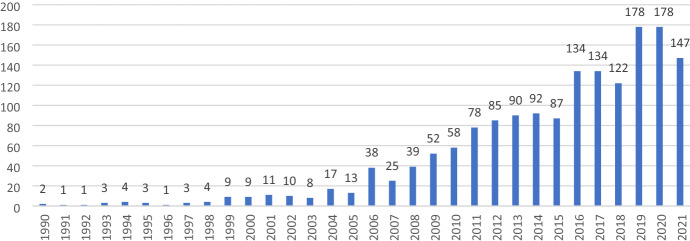
Number of articles published in EBSO and Scopus from 199 to 2021

**Fig. 2:**
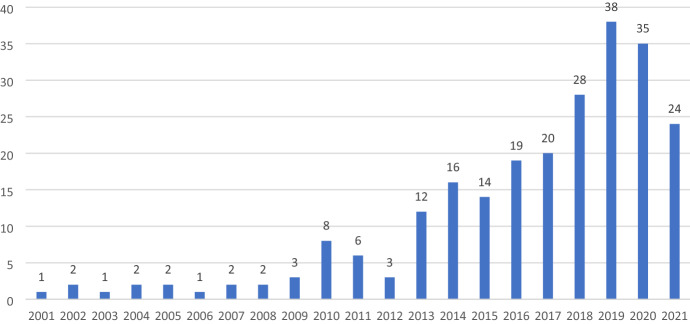
Number of articles published in Web of Science from 2001 to 2021

**Fig. 3:**
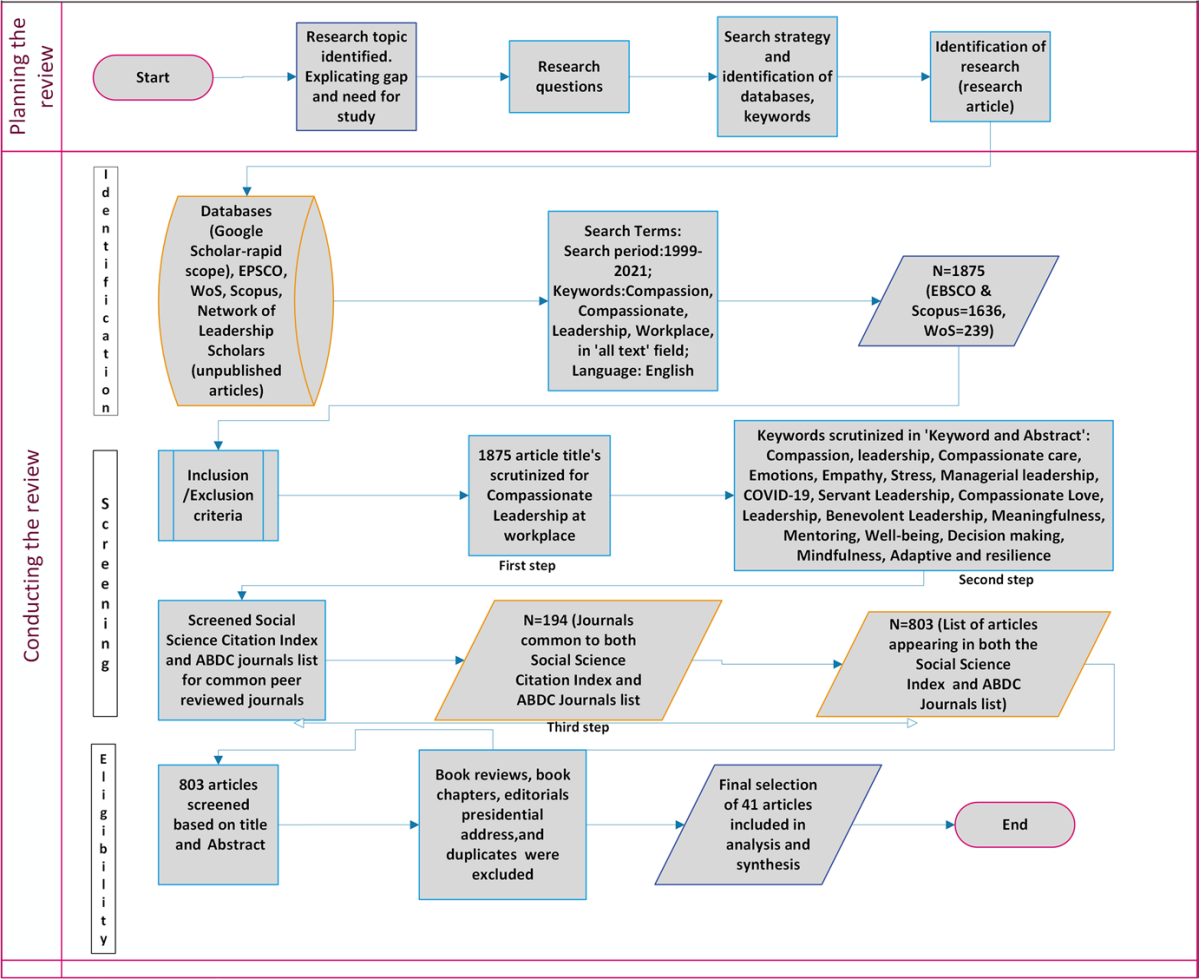
Flow diagram for Systematic Literature Review

1. Firstly, a cursory electronic search (rapid scope) through Google Scholar was conducted.
2. Secondly, databases such as EBSCO Discovery Services, Web of Science Core Collections and Scopus were searched. In addition, as recommended by Webster and Watson (2002) a search was performed to identify relevant papers from the citations of articles.
3. Thirdly, unpublished articles were solicited from a body of leadership scholars listed in the Network of Leadership Scholars through Connect. Aom.Org. Kuckertz and Block (2021) propose inclusion of more than one database to address triangulation which helps compensate for any disadvantage emanating from a single database.
4. The search scanned the years 1999–2021 as 1999 was the year when Frost’s first peer-reviewed call on compassion at workplace was published (Frost 1999). However, articles published before 1999 relating to compassionate leadership were also considered to trace the evolution of the concept.
5. As the topic is about compassionate leadership, the key words used were ‘compassion’, ‘compassionate’, leadership, ‘workplace’ in ‘all text’ field. The search was limited to English language studies, being the main language of the researchers.
6. The search in EBSCO and Scopus yielded a combined list of 1636 articles (Fig. 1). Likewise, a search in the Web of Science Core Collection database yielded a list of 239 articles (Fig. 2). No unpublished articles were made available from the Network of Leadership Scholars forum. Thus, a total of 1875 articles were identified (see Fig. 3).

### Inclusion/exclusion criteria

The inclusion and exclusion followed a four-step process.

1. In the first step, the titles of all retrieved articles (n = 1875) were scrutinized to identify the topic of compassionate leadership at workplace.
2. In the second step, the identified studies were scrutinized for the keywords such as *compassion, leadership, compassionate care, emotions, empathy, stress, managerial leadership, COVID-19, servant leadership, compassionate love, leadership, benevolent leadership, meaningfulness, mentoring, well-being, decision making, mindfulness, adaptive and resilience, narcissism.* These keywords are found to be used in the literature of compassionate leadership studies. Keywords were looked up in two fields- keyword and abstract (Khandker 2022)
3. In the third step, the list of articles (n = 1875)-we called it sample A- were reviewed to focus on articles published in all well recognized journals from 1999 as listed in the Social Science Citation Index or ranked in the Association of Business Deans Council (ABDC). In order to do that, we performed two comparisons. (1) Compared the journals in the Sample list A with the list of journals from the Social Science Citation Index and (2) compared the sample list A with the list of journals from the Association of Business Deans Council (ABDC). The list of journals in the Social Science Citation Index (updated as of 21st August 2021) was downloaded on 6th Sept 2021. Likewise, the list of journals in ABDC (updated as of 6th December 2019) was also downloaded on 6th Sept 2021. Since a manual comparison was difficult to perform, a free comparison tool located through an extensive internet search (https://www.xlcomparator.net/index.php) was identified and used. Using this tool, we first compared the Sample A list with the Social Science Citation index list and it identified a list of 174 common journals. Second, we compared the Sample A list with the list of ABDC journals list and it identified up a list of 222 common journals. The two lists comprising of 174 and 222 names of journals were then checked to identify any common journals in both the Social Science Citation index and the ABDC journals list. This yielded a total of 194 journals in both the databases. This list of 194 journals was used to identify the articles and it yielded a list of 803 articles.
4. In the final step, the abstracts from this list of 803 articles were reviewed by the authors to decide which article to include and a judgement was made based upon the theme of compassionate leadership. Book reviews, book chapters, editorials and a Presidential address were excluded from the selection. Duplicates were also removed. The reviewers also considered inclusion of some of the seminal and highly cited articles. The final list for review comprised of 41 articles. In line with the recommendations of Kuckertz and Block (2021) the selection process is illustrated as a flowchart in Fig. 3.

True to the interdisciplinary nature of leadership, compassionate leadership studies transcend several disciplines (Eva et al. 2019). The most dominant discipline in which compassionate leadership has been studied is that of healthcare (n = 17). On the other hand, studies in the discipline of Business/Management are a respectable number (n = 14) and seem to be a growing area of interest among the Business/Management scholars. Studies related to compassionate leadership have also emerged in other disciplines such as Organizational Science/Studies (n = 4), Political Science/Administration (n = 2), Family violence (n = 1), Physical education (n = 1), and Human Resource (n = 1).

Our review of the articles published reveals that a majority of these publications are mostly qualitative (n = 34) see (Table 1), while there are 5 articles which are quantitative (see Table 2) and 2 studies employing mixed methods approach (Table 3). Of the 41 studies, 27 were empirical (Table 4) while 14 were non-empirical (Table 5). The review reveals that compassionate leadership is still in a nascent stage. Even though the year 1999 was selected as the base year for identifying studies based on the theme of compassionate leadership, most of the studies emerge from 2002 with several important contributions from (Frost et al. 2004; Kanov et al. 2004; Lilius et al. 2003) in the form of commentary and discussions.

**Table 1:** Details of qualitative studies

S.No	Author/s	Research area	Research question	Epistemology/methodology	Method	Data	Data analysis	Findings	Contribution	Future research
1	Foster (2021)	Authors viewpoint on finding the right leadership style	Not available	Constructivism/Qualitative	Not available	Not available	Not available	Not available	The need for leaders to show respect, compassion and kindness towards their staff is emphasized	Not available
2	Papadopoulos et al. (2021)	Exploratory study on the views of healthcare managers for the definition, advantages and importance of compassion	(1) How is compassion defined? (2) What are the advantages of giving compassion to staff? (3) Why receiving compassion is important?	Social Constructivism/Qualitative	Exploratory Online Survey/Both open and close-ended questions	1217 respondents	Interpretive/Thematic analysis	Proposes a definition of compassion from the receivers’ point of view. Compassion is viewed as functional in establishing a trustworthy manager-staff relationship	Exploratory study on how healthcare managers view, define, recognize and practice culturally competent compassion	Future empirical study to include multidisciplinary, local and transnational collaborations
3	Jones et al. (2021)	The role of a Health, Social Work department in responding to the Pandemic with different initiatives	Not available	Not available	Qualitative/Descriptive	Secondary data. Department reports	Not available	Describes how integrating social work at the leadership level allows social workers to extend their expertise in community organizing, clinical care and compassionate communication to the community	Inclusion of social work leadership is proposed which would enable faster adaptation and organization of efforts to integrate psychosocial care and health care focused on equity and justice	Not available
4	Oruh et al. (2021)	Exploratory study on how compassionate managerial leadership style can mitigate workplace stressors and alleviate stress experiences among employees in an extreme situation	What does employee stress entail during the COVID-19 crisis, and how can an effective management-leadership mechanism help to reduce employee stress levels during such an extreme situation?	Interpretivism/Qualitative	Semi-Structured telephone interviews	30 participants	Thematic analysis	Four key themes are inductively derived; Conceptualizing compassionate care; Transference; Transforming and Sustainability	Study reveals participants view of compassionate care as holistic, empathetic, as well as caring for self and others Being able to 'stand in the patients’ shoes’ demonstrates empathy and compassionate care in clinical practice	Not available
5	Vogus et al. (2020)	Reviews empirical research to examine how a combination of interpersonal acts, leadership style and organizational structures underpins leading with compassion in the context of health services, management and medicine	(1) How leading with compassion encompasses three interrelated processes (2) How leaders implement practices and structures to systematically enable compassion and sustain a culture of compassion	Interpretivism/Qualitative	Literature review	Not available	Not available	Expression of compassion results in better experiences, outcomes for patients and meaningful, rewarding and sustainable work for care providers	Provides recommendations for leaders to cultivate a culture of compassion and compassionate care	Not available
6	König et al. (2020)	How top managements level of empathy affect their management of organizational crises	How does a CEO’s level of trait empathy affect his or her management of organizational crises?	Constructivism/Qualitative	Literature prompts discussion/Inductive reasoning	Not available	Not available	Not available	Theorizing and model development	Proposes empirical research to test the hypothesized relationships
7	Hofmeyer et al. (2020)	Presents a series of discussion papers on the relevance of empathic healthcare culture and constructs such as empathy; emotion regulation; compassion to sustain well-being; resilience and effectiveness during volatile times	Not available	Constructivism/Qualitative	Not available	Staff survey results	Not available	Not available	Present strategies to identify areas for organizational improvement, to promote engagement and reduce caregiver burnout	Not available
8	Mayer and Oosthuizen (2020)	Presents insights into salutogenesis particularly for leaders coping with challenges faced with the transition into the 4th industrial revolution	Series of questions referring to sense of coherence, comprehensibility, manageability, meaningfulness, coping, leadership and compassionate love	Hermeneutics/Qualitative	Qualitative structured questionnaires administered through email to respondents	22 respondents from various countries, cultural and religious backgrounds	Five-step process of content analysis. Deductive research interpretation process	The findings support previous studies on Sense of Coherence (SOC)as a coping mechanism to manage work stressors and challenges	Compassionate love is a strong coping mechanism connected with SOC and Coping	Not available
9	Hougaard et al. (2020)	Discussion about the importance of combining compassion with wisdom as a key component of compassionate leadership	Not available	Qualitative	Not available	Not available	Not available	Not available	Proposes combining compassion with wisdom for compassionate leadership Presents a wise compassionate leadership matrix	Not available
10	Landers et al. (2020)	Explores nurses’ and midwives’ views of a three-day program from the perspective of participating nursing/midwifery leaders, directors of nursing/midwifery, chief directors of nursing/midwifery, and program facilitators	(1) How compassionate care was conceptualized; (2) How participants viewed the delivery of the program? (3) How participants viewed the impact of the program on self and on practice? (4) How participants believed the program could be developed?	Interpretivism/Qualitative	Qualitative descriptive design/Semi-structured interviews	15	Thematic analysis	Four key themes are inductively derived; Conceptualizing compassionate care; Transference; Transforming and Sustainability	Participants view compassionate care as holistic, empathetic, as well as caring for self and others. Being able to 'stand in the patients’ shoes' demonstrates empathy and compassionate care in clinical practice	Not available
11	Hewison et al. (2019)	Explores compassionate leadership with those involved in leading system-wide end-of-life care	Stated as objectives of the research; To define compassionate leadership in the context of end-of-life care To collect accounts of compassionate leadership activity To identify examples of compassionate leadership in practice	Qualitative	Focus Group	14 participants with discussions lasting between 46 and 70 min	Thematic analysis using Porter's cyclical approach	Identifies participants viewpoints on what compassionate leadership is/should be Provides accounts and examples of compassionate leadership practices in the delivery of end-of-life care	Leadership in the Palliative and End-of-Life Care (PEoLC) needs to be understood in terms of practices and organizational interventions and not just based on personal behavioural style or competences	Not available
12	Shuck et al. (2019)	Study conceptualizing compassionate leader behaviour	(1) Is there a distinctive subset of leader behaviours related to the experience of compassionate leadership? (2) What is the influence of a compassionate leader behaviour on performance within the framework of HRD?	Phenomenology/qualitative	A two-stage, sequential, and equal status mixed method research design; Semi-structured interviews	Open reflection and dialog from 22 leaders formed the basis of the data	Thematic analysis using the methodology proposed by Lilius et al. (2011)	Six distinctive themes—integrity, empathy, accountability, authenticity, presence, and dignity—are identified as individual-level building blocks of compassionate leadership behaviour	Develops and validates a Compassionate Leader Behaviour Index (CLBI) based on six emergent behaviours	Research using a diary study methodology to closely examine the nuances of compassion alongside theories of leadership Research using Longitudinal and experience-based sampling techniques coupled with larger samples Research to explore, test and validate the model proposed by the authors Research to consider the application of compassionate leader within a cross section of industries and cultures
13	Lown et al. (2019)	Examines how legislative changes related to healthcare delivery influences health professionals’ capacity to offer compassionate care	Not available	Qualitative	Telephone surveys	Views of 500 physicians and nurses in 2010 and 2017	Compared and validated using Schwartz 12 item compassionate care scale	Compassion is inversely correlated with burnout	Proposes leaders must define the organizational conditions and implement processes that support professional’s innate compassion rather than addressing burnouts	Not available
14	Tzortzaki (2019)	Case study focusing on the need for young adults to develop compassionate self-leadership competencies to thrive and be of service in today's fast paced polarized world	Not available	Qualitative/Case study	Two qualitative questionnaires: (1) An end of semester evaluation, (2) A pre and post compassionate self-leadership questionnaire	78 participants	Paired Samples t-test to compare scores of the participants at the beginning and end of the course	Students could potentially improve their level of compassionate self-leadership by undergoing a course	Proposes a conceptual framework of compassionate self-leadership	Not available
15	Willis and Anstey (2019)	Case study of compassionate leadership in the context of palliative care	Not available	Qualitative	Single case study	Reflective notes from the case of a single patient requiring palliative care	Not available	Compassionate leadership can ensure that the value of staff wellbeing is acknowledged and, patients would feel that they are being cared for and not ‘just treated’. Compassionate leadership would also work well within the ‘quasi-family’ model of leadership	Not available	Not available
16	Hewison et al. (2018)	Study evaluating a compassion recognition scheme	What helped and/or hindered the roll out of the scheme?	Qualitative	Semi-structured interviews and a focus group discussion	8 staff members from 10 participating organizations A focus group consisting of 3 participants Accounts of compassionate acts from 1500 nomination forms	Thematic analysis of the accounts of compassionate acts from the 1500 nominations	Seven action and impact themes resulting in a framework of compassion	Proposes the first typology of compassion	Not available
17	Hougaard et al. (2018)	Questionnaire instrument development for compassionate leadership	Not available	Qualitative	Not available	Not available	Not available	Not available	Proposes a measurement instrument for assessing compassionate leadership	Not available
18	Banker and Bhal (2018)	Examines how practicing managers construe the concept of compassion in business organizations	(1) How is compassion construed by the managers? (2) Does compassion prevail in their organization or not?	Interpretivism/Qualitative	Qualitative In-depth interviews in person or telephone lasting 20–30 min. Semi-structure open-ended questions	10 practicing managers	Grounded theory approach/Used axial coding	Presents the views of participants from two different lenses; (1) Receiver (2) Giver Provides the factors responsible for both compassion and non-compassionate organizations	-Provides an understanding of compassion purely from a business organizations point of view Proposes the view that organizations where compassion is absent should be called the 'absence of compassion' rather than being coloured negatively Proposes a need for empathy in leaders to create a compassionate organization	To apply and recheck the proposed framework for an international audience A research based on real aspects of leadership rather than on perceptions
19	MacArthur et al. (2017)	A critical analysis of a 'Leadership in Compassionate Care (LCC) Programme’	Four research questions: (1) What are the views, experiences and perceptions of participating stakeholders of the impact of the LCC Programme? (2) How are the mechanisms used in the LCC Programme seen to influence the outcomes in different clinical settings? (3) What are the early signs of sustainability of the LCC Programme? (4) What factors can be drawn into a conceptual model for enhancing organisational capacity to develop and sustain a culture of compassionate care	Realism/Qualitative	Longitudinal study Semi-structure interviews/Informal observation/Attendance at meetings/Review of research outputs	26 participants; 39 Structured interviews, 3 focus groups lasting from 57 min to 2 h	Thematic analysis/Realistic evaluation framework. Data coded and managed in NVivo 9	Generated a dynamic, practice-based model for strengthening organisational capacity for compassionate care	Adds to a body of work of evidences on what compassionate care means to different stakeholders	Research on identifying the organizational infrastructure needed to embed and sustain a focus on compassionate care
20	Milner (2017)	Essay on embracing compassion in community development and social action	Not available	Qualitative/Descriptive	Not available	Not available	Not available	Relational community development practice driven by compassion enhances social trust, fairness and justice	Not available	Not available
21	de Zulueta (2016)	An integrated literature review about compassion from different perspectives including neuro science; psychology, complexity science, modern leadership and organizational theories	Not available	Qualitative	Literature review	Review of articles	Not available	Not available	Recommends a paradigm shift from an engineering hierarchical model of organizations with trait-based, top-down individualistic models of leadership toward a model of the organization as a complex living system and leadership as adaptive, shared, and distributed	Not available
22	Wei et al. (2016)	Study about compassion from a Confucian theory perspective complementing western theory	What are the antecedents for self-moral development to initiative compassionate action? What role do self-moral development play in shaping organizational outcomes and leadership in the practice of compassion?	Qualitative	Grounded theory Critical incident interviews	On site interviews with 31 senior executives from four cities in North China	Grounded theory approach/Open and axial coding	Integrating compassion and self-cultivation enriches an understanding of the moral growth of compassion Compassion affects performance outcomes at individual, relational and organizational level Self-cultivation and compassion have an ultimate interaction effect on organizational outcomes	Contributes to compassion research from the perspective of indigenous theory to complement western theory Develops an integrative framework for understanding compassion	Research to examine the antecedents for self-moral development to initiative compassionate action? What role do self-moral development play in shaping organizational outcomes and leadership in the practice of compassion?
23	Dewar and Cook (2014)	An evaluation of a leadership development programme in a health care facility in Scotland	Not available	Qualitative	Questionnaire and semi-structured interviews	86 nursing staff covering 24 in patient areas	Immersion crystallisation technique and Descriptive statistics	Not available	Not available	Not available
24	Seco and Lopes (2014)	Develops a structure for trust building in an educational context	Not available	Qualitative	Grounded theory/Open-ended questionnaire	12 Principals from 44 schools in Portugal	Open Coding/Axial coding/Selective Coding	Proposes compassion to be complemented with assertiveness to become effective	Contributes to studies on trust relations and identifies two dimensions: Compassion and assertiveness to become effective	Not available
25	Karakas and Sarigollu (2013)	Examines the role of benevolent leadership in creating a virtuous and compassionate organization	What was it like for you to experience compassion at work? Can you tell a specific example?	Phenomenology/Qualitative	Narrative Inquiry/Open ended questions/Semi Structured interviews	32 Semi-structured interviews with managers, employees and other stakeholders	Interpretive Phenomenological Analysis. Uses a predetermined benevolent framework for the narrative analysis	Develops conceptual model of benevolent leadership based upon four inter-related streams of research	Contributes to advancement of virtue ethics scholarship and practice	Research to develop psychometric, experimental, and ethnographic methods for further exploration, opera- tionalization, and measurement of benevolent leadership in organizations Empirical research to refine the construct of benevolent leadership Research to address how different leadership styles and roles interrelate and complement one another to create compassionate and virtuous organizations
26	McKimm and O’Sullivan (2013)	Discussion paper on a published report	Not available	Qualitative/Descriptive	Not available	Not available	Not available	Clinical leadership and doctors’ engagement in management is vital to maintain the focus on providing compassionate care to all	Not available	Not available
27	Hornett (2012)	Viewpoint on compassionate leadership	Not available	Qualitative/Descriptive	Not available	Not available	Not available	Not available	Stresses upon the importance of integrating compassionate care into the work	Not available
28	Rynes et al. (2012)	Editors’ reflections on a Special Issue on Understanding and Creating Caring and Compassionate Organizations	Not available	Qualitative/Descriptive	Review of articles selected for the special issue	9 articles	Not available	Not available	Points out to the rich learnings management research can draw upon from the social science studies on care and compassion	Not available
29	Dutton and Workman (2011)	Essay on how compassion is a generative force drawing upon the work of Frost (1999)	Not available	Qualitative/Descriptive	Narrative analysis	Several compassion stories	Not available	Elaborates on how compassion as a generative force opens up new vistas, expands resources and creates new insights	Builds upon the work of Frost (1999) and highlights how research into compassion has grown since Frost's seminal call	Not available
30	Martinek et al. (2007)	Describes the evolution of youth leadership from two education programs serving low-income minority youth	Not available	Qualitative/Descriptive	Case study	Interviews and written reflections of participants	Case study analysis	The ability of adolescents to progress through different stages of leadership is related to their personal needs their levels of moral development	Not available	Not available
31	Boyatzis et al. (2006)	Study integrating findings in affective neuroscience and biology with research on leadership and stress	Not available	Qualitative	Not available	Based on recent studies in affective neuroscience, biology and studies on leadership and stress	Integrative review	Demonstration of compassion through coaching others may increase leaders’ sustainability by reducing the effects of power stress Leaders are more likely to be effective and resist the effects of chronic power stress if they are physiologically and psychologically balanced through the act of coaching others	Proposes incorporation of psychophysiological interactions for a holistic leadership and leadership development	Longitudinal studies to support or refute the theoretical propositions presented Studies to consider physiological measures when conducting leadership or leadership development studies Interdisciplinary research on leadership Experiments to discover the degree to which a person feels better, experiences compassion and activates their parasympathetic nervous systems Epidemiological studies of leaders with historical records and interviews
32	Dutton et al. (2006)	Study on how individual compassion is coordinated through a process called compassion organizing	Three research questions: (1) What was the pattern of action prompted by the fire? (2) What roles did individuals play? and (3) What features of the organization enabled or disabled the pattern of response?	Interpretivism/Qualitative	Grounded theory/case study	Interviews, archival electronic correspondence and audience responses to a case Fifteen interviews, archival records from the Deans office and response from 65 MBA students to a case	Thematic analysis	Compassion organizing is a joint product of structures of an organization; the agency of individuals who get engaged in the process; and emergent features. The three features form the skeleton of a theory of compassion organizing The theory implies that organizations cannot create compassion by simply hiring compassionate people The theory also implies that compassion organizing cannot be achieved by having compassionate leaders. Rather leaders’ actions can contribute to compassion organizing by strengthening features of social architecture	Theory development and offers a definition of compassion organizing.	Research on how features of an organizational context encourage and enable emotional expression, public emotional displays, and emotion-based responses contributing to compassion organizing. Research on how social resources such as trust and legitimacy are enabled by organizational routines, values, and networks and how they fuel the processes that allow organizations to respond adaptively to triggers beyond the trigger of human pain.
33	Kanov et al. (2004)	Exploratory study on the process of compassion in organizations	Not available	Interpretivism/Qualitative	Conceptual	Not available	Not available	Process of compassion is more than an individual experience and in which a number of people individually engage	Conceptualizes organizational compassion Contributes to the field of positive organizational studies by identifying a number of systemic features through which an organization can increase their capacity for organizational compassion	To examine how members of organization can create pockets of collective compassion within an organization An empirical investigation examining the combination of organizational features most important in propagating, legitimating, and coordinating different sub processes of compassion An ethnography study to observe and engage with organizational members at close quarters and over time
34	Frost (1999)	Reflective essay on the importance of compassion in the understanding of organizational life	Not available	Qualitative/Descriptive	Discussion of articles on compassion, theory building and relational practice	4 articles	Not available	Identifies four tracks relating to compassion, theory building and relational practice	Seminal contribution on understanding organizational life through the lens of compassion	Research into how compassion could address the suffering & pain, the toxicity prevalent in organizations and to look at organizational life through the eyes of compassion

**Table 2:** Details of quantitative studies

S.No	Author/s	Research area	Research question	Epistemology/Methodology	Method	Data	Data analysis	Findings	Contribution	Future research
1	Brouns et al. (2020)	Study examining whether leader's propensity for compassionate love evokes servant leadership behaviour	Two Hypotheses: (H1) Leaders compassionate love towards non-intimate others is positively associated with servant leadership behaviour; (H2) Leader’s narcissism is negatively associated with servant leadership behaviour	Quantitative/Descriptive statistics	Cross-sectional survey through online questionnaire	170 respondents	Confirmatory analysis, OLS Regression analysis	Leaders’ self-assessment of compassionate love was positively co-related with the servant leadership behaviour as perceived by the employees Narcissism was negatively associated with servant leadership Compassionate love is positively associated with servant leadership	First empirical study in support of a theory that compassionate love is a cornerstone of servant leadership	A study to understand which variable accounts for the largest variance in servant leadership To understand the situation variables that promote servant leadership
2	Paakkanen et al. (2021)	Examines whether compassion could be increased among managers through improving their emotional skills	Six hypotheses to test whether the training intervention would have positive impacts	Quantitative	Randomized quasi-controlled trial of a new in-depth emotional skills cultivation training versus a control condition	Sixty-eight treatment group participants and 90 control group participants. Additional four intervention groups participated. Eighty-five treatment group followers and 72 control group followers Data was collected from Self-reported questionnaires from managers and their followers a week before and a week after training	Confirmatory factor analysis	Emotional Skills Cultivation Training (ESCT) was related to significant improvement in managers sense of compassion Emotional skills could improve compassion. Servant leadership showed signs of improvement after the training	Contributes to literature on compassion in organizations, leadership and compassion intervention by suggesting that compassion can be increased in organizations among managers through emotional skills and by introducing a new compassion-inducing emotional skills intervention and a new emotional skills measure	Longitudinal research to study whether the positive impacts of compassionate acting can be sustained over time Study about potential factors that might facilitate or hinder long-term effect of the training intervention Carefully designed randomized controlled trials with a placebo or an active compassion group Research to develop separate scales for each sub dimension of compassion and include additional methods of examining changes in compassion Study to identify the most effective modules & types of trainings to build effective compassion trainings
3	Basran et al. (2019)	Studies two types of leadership styles: Antisocial and Prosocial	Set of hypotheses hypothesizing antisocial and pro-social leadership styles to be co-related with several dimensions of these two leadership styles	Quantitative	A set of self-report scales administered to students of a university	219 student respondents	Predictive analysis software (PASW) and Analysis of Momentary structure (AMOS); Series of Pearson Product Moment Correlation; a Principal Component Analysis; Multiple linear regression; and Path analysis	Antisocial leadership is fearful of offering compassion to others and resistance to compassion. Prosocial leadership is associated with coalition building, secure striving, compassionate goals and feeling socially safe	Not available	Cross-sectional research to look at individuals in leadership positions Study to generate new measures designed to compare and contract prosocial and antisocial leadership The role of gender variation on the two dimensions of leadership- prosocial and antisocial interpersonal styles The role of variables like empathy, emotion regulation and moral reasoning in relation to the leadership to the two leadership styles
4	Zoghbi-Manrique-de-Lara and Viera-Armas (2019)	Study about compassionate feelings as mediators of the relationship between ethical leadership and interpersonal citizenship behaviour directed at peers	Four hypotheses about compassionate feelings	Quantitative	Questionnaires emailed to leaders of 100 banks in London in 2015	Data collected from 300 work colleagues at 100 banks. A 7-point Likert-type scale and a 10-item measure were employed to examine the different hypotheses	Data was analysed using SPSS, structural equation modelling. AMOS 22 was used for confirmatory factor analyses. Hayes approach was used to test mediation	The study confirms that when staff are recipients of acts of ethical leadership, they respond to this with peer-focused Organizational Citizenship (OCB) Behaviours empathic concern, mindfulness, and kindness play an insignificant role	Contributes to developing the literature on the impact of ethical leadership on followers’ ethical helping behaviour	A study using data triangulation to gain more qualitative insights Research to include specific impacts on the perception of culture and ethical leadership Research to include corporate culture, ethical leadership, and peer-focused Organizational citizenship behaviours (OCBs) Whether perceptions of ethical leadership could vary depending on the different areas or services employee’s work
5	Eldor (2018)	Investigates the effect of compassion in the public service workplace	Four hypotheses about compassion and its association with public service employees work engagement, work burnout, their OCB performance and knowledge sharing and their compassionate oriented behaviour towards clients	Quantitative	Longitudinal study	Data was collected from a major public service organization in Israel in a 6 months’ time difference Three sources of questionnaires were distributed to three different data sources. A final sample of 166 completed the questionnaires	Used HLM to test the hypotheses. Used guidelines from previous studies for probing moderating effects. Other indices used include the comparative fit index (CFI), the normed fit index (NFI), the root mean square error of approximation (SRMR)	Compassion is of undeniable importance in the public sector which stimulates significant attitudinal outcomes, such as work engagement and work burnout, intensifies OCB and knowledge-sharing Receipt of compassion from supervisors has a contagious effect and impacts compassionate employee behaviour	Contributes to the study of compassion in public management literature	Research to include variables such as quality of service and client satisfaction to study the effect of compassion on the service domain Research to expand on the proposed model by including the mediators of social exchange, LMX transaction and trust building experiences Research into the role of compassion in cultivating healthier interactions between public service agencies and citizens Research clarifying the interrelated relationship between compassion and other public sector concepts such as OCB, prosocial behaviour, and emotional labour

**Table 3:** Details of mixed method studies

S.No	Author/s	Research area	Research question	Epistemology/Methodology	Method	Data	Data analysis	Findings	Contribution	Future research
1	Coffey et al. (2019)	Systematic review on the impact of compassionate care education programmes on nurses	(1) What is the impact of compassionate care education programmes on registered nurses (RNs), clinical nursing leaders, nursing educators, and/or nursing students? (2) What programme characteristics have led to positive and/or negative outcomes? and (3) What are the barriers and/or facilitators to the implementation of compassionate care education programmes?	Pragmatism/Mixed Methods	Mixed Methods PICOS framework guided the database search Quality was appraised using Critical Appraisal Skills Programme Checklist; Mixed -Method Appraisal Tool and the Effective Practice and Organization of Care tool	Used Covidence to export and screen the articles. A total of 15 papers were identified for review from 551 records	Uses Parallel-results convergent synthesis	Six sub-themes identified from the synthesis Compassionate care programmes had a positive effect on clinical leadership and confidence to lead change in practice compassionate care education led to improved job satisfaction, heightened sense of well‐being, and increased pride in the nursing profession	Stresses a strong need to establish novel education programmes to promote compassionate care and self-compassion among nursing staff	Research to include a 360-degree evaluation of educational programmes in compassionate care Longitudinal study to explore the long-term impact of compassionate care programmes on patient outcomes and outcomes in relation to leadership building skills, and to assess whether positive outcomes were maintained over time Study using valid and reliable data collection instruments and representative samples
2	Lilius et al. (2008)	Study about compassion at work from two angles	Three hypotheses: Hypothesis 1. There will be a positive relationship between experienced compassion at work and the frequent inducement of positive emotion at work Hypothesis 2a. There will be a positive relationship bet ween experienced compassion at work and affective organizational commitment Hypothesis 2b. There will be a positive relationship between experienced compassion at work and affective organizational commitment through the frequent inducement of positive emotion at work	Pragmatism/Mixed Methods	Mixed Methods: Pilot survey and Narrative Inquiry	Both Quantitative and Qualitative: 239 survey respondents from a hospital in the Central US 171 stories of compassion at work from respondents	Structural Equation Modelling using the EQS Program Used coding for basic narrative characteristics and a counting and coding strategy from content analysis research Used Cohen's Kappa to measure the inter-rater reliability	Experiencing compassion at work is important for positive emotion and affective organizational commitment Effect of experienced compassion accumulates to leave a lasting trace on organization members Compassion is more frequently received from co-workers than from supervisors unlike research indicating the importance of supervisors as a source of support Compassion is experienced with equal frequency among those who do and do not manage other employees Compassion is least frequent among those involved in the 'business of care' Three major categories of compassion at work emerge (1) giving emotional support, (2) giving time and providing flexibility, and (3) giving material goods Experiencing compassion indirectly may be as powerful as receiving it Positive emotion mediates the relationship between experienced compassion and other important workplace outcomes Employees use instances of compassion to make sense of their own ability to contribute and carry on their work, and also to understand their co-workers and their work environment Absence of compassion can have a significant impact on employees' construction of their co-workers and their overall work environment	(1) Contributes to the stream of studies depicting organizations as relational systems and sites of social life (2) Contributes to the body of research on positive emotion in an organizational setting than laboratory based (3) Contributes to previous work that advocates the inclusion of 'emotional sense' in the discussion of sensemaking in organizations (4) Contributes to studies in the field of positive organizational behaviour and positive organizational scholarship by pursuing a deeper understanding of the conditions that foster positive states in work organizations	Research into how different organizational contexts might facilitate or hinder the expression of compassion Research that addresses the influence of occupational and organizational culture in the enactment of compassion at work How significant are events outside the organizational policies and practices in producing a compassionate response? To explore the link between compassion and other organizational outcomes such as absenteeism, turnover and recovery time To examine compassion from an angle of normative component from the view of organizational commitment

**Table 4:** Details of empirical studies

S.No	Author/s	Research area	Epistemology/methodology	Method	Data	Data analysis
1	Martinek et al. (2007)	Describes the evolution of youth leadership from two education programs serving low-income minority youth	Qualitative/Descriptive	Case study	Interviews and written reflections of participants	Case study analysis
2	Dutton et al. (2006)	Theoretical study on how individual compassion is coordinated through a process called compassion organizing	Interpretivism/Qualitative	Grounded theory Case study	Interviews, archival electronic correspondence and audience responses to a case Fifteen interviews, archival records from the Deans office and response from 65 MBA students to a case	Thematic analysis
3	Lilius et al. (2008)	Study about compassion at work from two angles	Pragmatism/Mixed Methods	Mixed Methods: Pilot survey and Narrative Inquiry	Both Quantitative and Qualitative: 239 survey respondents from a hospital in the Central US 171 stories of compassion at work from respondents	Structural Equation Modelling using the EQS Program Used coding for basic narrative characteristics and a counting and coding strategy from content analysis research Used Cohen's Kappa to measure the inter-rater reliability
4	Dutton and Workman (2011)	Essay on how compassion is a generative force. Draws upon the work of Frost (1999)	Qualitative/Descriptive	Narrative analysis	Several stories related to compassion	Not available
5	Karakas and Sarigollu (2013)	Examines the role of benevolent leadership in creating a virtuous and compassionate organization	Phenomenology/Qualitative	Narrative Inquiry/Open ended questions/Semi structured interviews	32 Semi-structured interviews with managers, employees and other stakeholders	Interpretive Phenomenological Analysis. Uses a predetermined benevolent framework for the narrative analysis
6	Dewar and Cook (2014)	An evaluation of a leadership development programme implemented in a health care facility in Scotland	Qualitative	Questionnaire and semi-structured interviews	86 nursing staff covering 24 in patient areas in Health Care facility in Scotland	Immersion crystallisation technique and Descriptive statistics
7	Seco and Lopes (2014)	Develops a structure for trust building in an educational context	Qualitative	Grounded theory/Open-ended questionnaire	12 Principals from 44 schools in Portugal	Open coding/Axial coding/Selective coding
8	Wei et al. (2016)	Study about compassion from a Confucian theory perspective complementing western theory	Qualitative	Grounded theory Critical incident interviews	On site interviews with 31 senior executives from four cities in North China	Grounded theory approach/Open and axial coding
9	MacArthur et al. (2017)	A critical analysis of a 'Leadership in Compassionate Care Program’	Realism/Qualitative	Longitudinal study Semi-structure interviews/Informal observation/Attendance at meetings/Review of research outputs	26 participants; 39 structured interviews, 3 focus groups lasting from 57 min to 2 h	Thematic analysis/Realistic evaluation framework. Data coded and managed in NVivo 9
10	Hewison et al. (2018)	Evaluation of a ‘Leading with compassion recognition’ scheme	Qualitative	Semi-structured interviews, a focus group	8 staff members from 10 participating organizations A focus group consisting of 3 participants Accounts of compassionate acts from 1500 nomination forms	Thematic analysis of the accounts of compassionate acts from the 1500 nominations
11	Eldor (2018)	Investigates the effect of compassion at a public service workplace	Quantitative	Longitudinal study	Data from a major public service organization in Israel in a 6 months’ time difference Three sources of questionnaires were distributed to three different data sources. A final sample of 166 completed the questionnaires	Used HLM to test the hypotheses. Used guidelines from previous studies for probing moderating effects. Other indices used include the comparative fit index (CFI), the normed fit index (NFI), the root mean square error of approximation (SRMR)
12	Banker and Bhal (2018)	Examines how practicing managers construe the concept of compassion in business organizations	Interpretivism/Qualitative	Qualitative In-depth interviews in person or telephone lasting 20 to 30 min. Semi-structure open-ended questions	10 practicing managers	Grounded theory approach/Used axial coding
13	Shuck et al. (2019)	Conceptualization of compassionate leader behaviour	Phenomenology/Qualitative	A two-stage, sequential, and equal status mixed method research design Semi-structured interviews	Open reflection and dialog from 22 leaders formed the basis of the data	Thematic analysis using the methodology proposed by Lilius et al. (2011)
14	Lown et al. (2019) (based on abstract)	Examines how legislative changes related to healthcare delivery influences health professionals’ capacity to offer compassionate care	Quantitative	Telephone surveys	Views of 500 physicians and nurses in 2010 and 2017	Compared and validated using Schwartz 12 item compassionate care scale
15	Tzortzaki (2019)	Case study focusing on the need for young adults to develop compassionate self-leadership competencies to thrive and be of service in today's fast paced polarized world	Qualitative/Case study	Two qualitative questionnaires: (1) An end of semester evaluation, (2) A pre and post compassionate self-leadership questionnaire	78 participants	Paired Samples t-test to compare scores of the participants at the beginning and end of their course
16	Willis and Anstey (2019)	Case study of compassionate leadership in the context of caring for a patient with a complex malignant wound needing palliative care	Qualitative	Single case study	Reflective notes from the case of a single patient requiring palliative care	Not available
17	Basran et al. (2019)	Studies two types of leadership styles: Antisocial and Prosocial	Quantitative	A set of self-report scales administered to students of a university	219 students	Predictive analysis software (PASW) and Analysis of Momentary structure (AMOS); Series of Pearson Product Moment Correlation; a Principal Component Analysis; Multiple linear regression; and Path analysis
18	Zoghbi-Manrique-de-Lara and Viera-Armas (2019)	Study about compassionate feelings as mediators of the relationship between ethical leadership and interpersonal citizenship behaviour directed at peers	Quantitative	Questionnaires emailed to leaders of 100 banks in the city of London in 2015	Data collected from 300 work colleagues at 100 banks. A 7-point Likert-type scale and a 10-item measure were employed to examine the different hypotheses	Data was analysed using SPSS, structural equation modelling. AMOS 22 was used for confirmatory factor analyses. Hayes approach was used to test mediation
19	Coffey et al. (2019)	Systematic review on the impact of compassionate care education programmes on nurses	Pragmatism/Mixed Methods	Mixed Methods PICOS framework guided the database search	Used Covidence to export and screen the articles. A total of 15 papers were identified from 551 records	Uses Parallel-results convergent synthesis
20	Hewison et al. (2019)	Explores compassionate leadership with those involved in leading system-wide end-of-life care	Qualitative	Focus Group	14 participants in the focus group discussions which lasted between 46 and 70 min	Thematic analysis using Porter's cyclical approach
21	Landers et al. (2020)	Explores nurses’ and midwives’ views of a three-day program entitled “The Leaders for Compassionate Care Program” from the perspective of participating nursing/midwifery leaders, directors of nursing/midwifery, chief directors of nursing/midwifery, and program facilitators	Interpretivism/Qualitative	Qualitative descriptive design/Semi-structured interviews	15 participants	Thematic analysis
22	Hofmeyer et al. (2020)	Discussion paper on the relevance of empathic healthcare cultures and constructs such as empathy, emotion regulation, compassion to sustain well-being, resilience and effectiveness during volatile times	Constructivism/Qualitative	Not available	Staff survey results	Not available
23	Mayer and Oosthuizen (2020)	Presents insights into salutogenesis particularly for leaders coping with challenges faced in the transition into the 4th industrial revolution	Hermeneutics/Qualitative	Qualitative structured questionnaires administered through email to respondents	22 respondents from various countries, cultural and religious backgrounds	Five-step process of content analysis. Deductive research interpretation process
24	Brouns et al. (2020)	Examines whether leader's propensity for compassionate love evokes servant leadership behaviour	Quantitative/Descriptive statistics	Cross-sectional survey through online questionnaire	170 respondents	Confirmatory analysis, OLS Regression analysis
25	Paakkanen et al. (2021)	Examines whether compassion could be increased among managers through improving their emotional skills	Quantitative	Randomized quasi-controlled trial of a new in-depth emotional skills cultivation training versus a control condition	Sixty-eight treatment group participants and 90 control group participants. Additional four intervention groups participated. Eighty-five treatment group followers and 72 control group followers. Data was collected from Self-reported questionnaires from managers and their followers a week before and a week after training	Confirmatory factor analysis
26	Papadopoulos et al. (2021)	Explore the views of healthcare managers to the definition, advantages and importance of compassion	Social Constructivism/Qualitative	Exploratory Online Survey/Both open and close-ended questions	1217 respondents	Interpretive/Thematic analysis
27	Oruh et al. (2021)	Exploratory study on how compassionate managerial leadership style can mitigate workplace stressors and alleviate stress experiences among employees in an extreme situation	Interpretivism/Qualitative	Semi Structured telephone interviews	30 participants	Thematic analysis

**Table 5:** Details of non-empirical studies

S.No	Author/s	Research area	Epistemology/methodology	Method	Data	Data analysis
1	Frost (1999)	Reflective essay on the importance of compassion in the understanding of organizational life	Interpretivism/Qualitative	Discussion paper	4 articles	Not available
2	Kanov et al. (2004)	Exploratory study on the process of compassion in organizations	Interpretivism/Qualitative	Conceptual	Not available	Not available
3	Boyatzis et al. (2006)	Study integrating findings in affective neuroscience and biology with research on leadership and stress	Interpretivism/Qualitative	Not available	Based on recent studies in affective neuroscience, biology and studies on leadership and stress	Integrative review
4	Hornett (2012)	Authors iewpoint on compassionate leadership	Qualitative/Descriptive	Not available	Not available	Not available
5	Rynes et al. (2012)	Editors’ reflections on a Special Issue on Understanding, Creating, Caring and Compassionate Organizations	Qualitative/Descriptive	Review of articles selected for the special issue	9 articles selected for the special issue	Not available
6	McKimm and O’Sullivan(2013)	Discussion paper on a published report	Qualitative/Descriptive	Not available	Not available	Not available
7	de Zulueta (2016)	An integrated literature review about compassion from different perspectives including neuro science; psychology, complexity science, modern leadership and organizational theories	Qualitative	Literature review	Review of many articles	Not available
8	Milner (2017)	Essay on embracing compassion in community development and social action	Qualitative/Descriptive	Not available	Not available	Not available
9	Hougaard et al. (2018)	Develops an assessment questionnaire for compassionate leadership	Qualitative	Not available	Not available	Not available
10	Vogus et al. (2020)	Reviews empirical research to examine how a combination of interpersonal acts, leadership style and organizational structures underpins leading with compassion in the context of health services, management and medicine	Interpretivism/Qualitative	Literature review	Not available	Not available
11	Hougaard et al. (2020)	Discussion about the importance of combining compassion with wisdom as a key component of compassionate leadership	Qualitative	Not available	Not available	Not available
12	König et al. (2020)	How top managements level of empathy affect their management of organizational crises	Constructivism/Qualitative	Literature prompts discussion/Inductive reasoning	Not available	Not available
13	Foster (2021)	Viewpoint on finding the right leadership style	Constructivism/Qualitative	Not available	Not available	Not available
14	Jones et al. (2021)	The role of a Health Social Work department in responding to the Pandemic with different initiatives	Qualitative/Descriptive	Review of department reports	Not available	Not available

## Findings

The findings are organized in line with the research questions.

### Definitions of compassionate leadership (RQ1)

In this section, we review how compassionate leadership has been defined in the selected articles and how compassionate leadership is understood. Literature on compassion reveals a variety of ways in which compassion has been defined, and most of the authors agree that compassion in leadership is an essential trait and the need of the hour, but one hardly finds an article which defines compassionate leadership clearly. Banker and Bhal (2018) associate compassionate leadership to a mindset which helps improve transcultural work relationships and understand complex situations, while Boyatzis et al. (2006) emphasizes the importance of coaching with compassion for leaders to sustain themselves in their role. Boyatzis et al. (2006) define coaching with compassion as coaching others with a view for their development unlike coaching which is strictly for the benefit of the organization. Vogus et al. (2020) associate compassionate leadership style to servant leadership which in their view is' inherently' compassionate. Delving a bit further, Brouns et al. (2020) attempt to understand whether compassionate love is an antecedent to servant leadership and find a positive association between a leaders’ compassionate love and servant leadership. Hewison et al. (2019) attempt to define compassionate leadership and find support in Vogus et al. (2020). They conclude that compassionate leadership is related both to servant leadership and resonant leadership combining the values of empathy, needs awareness, authentic response and commitment to growth of people. Oruh et al. (2021) offer the view that a managerial leadership -which combines the skills of a manager with the qualities of a leader- needs to include compassionate characteristics in order to be effective. They propose that a compassionate managerial leadership embodies an act of love, care and selflessness which enables a caring and supportive organization. Hougaard et al. (2020) assert that demonstrating compassion is not enough for leaders, for effective leadership, compassion must be combined with wisdom. They term this as wise compassionate leadership wherein leaders balance their concern for their employees with the need to move their organization forward in an efficient and productive manner. They assert that wise compassionate leadership enables tough action to be taken while genuinely caring for people's feelings and wellbeing. Paakkanen et al. (2021) find that compassion could be increased among managers through improving their emotional skills, unlike the traditional belief that compassion is an inherent trait. Coffey et al. (2019) present the view that effective leadership is the key to delivering safe and compassionate health care while stressing that lack of compassionate leadership results in a negative impact on organizational outcomes and quality. However, the study does not offer any definition of compassionate leadership.

Shuck et al. (2019) come very close to defining compassionate leadership when they conceptualize compassionate leader behavior as a new leadership construct embodying six behaviors- integrity, empathy, accountability, authenticity, presence, and dignity. Their study finds that these six behaviors are more likely to influence individual and organizational outcomes but they stop short of finding how much the impact would be on productivity, workplace culture, and firm performance. Tzortzaki (2019) propose that the core element of being a compassionate leader lies in them being self-compassionate. They assert that one cannot be an effective manager without being first and foremost effective at self-management. In other words, one cannot be compassionate towards others if one does not have the ability to be self-compassionate. Willis and Anstey (2019), drawing on a case of patient care, find support for four key elements proposed by West et al. (2017), which comprise of attending; understanding; being empathetic, and helping. By reviewing studies related to compassionate leadership in healthcare, de Zulueta (2016) stresses that the qualities of servant leadership, such as altruism; integrity; humility, and wisdom, combined with the qualities of appreciation and empowerment as required characteristics of compassionate leadership. Wei et al. (2016) emphasize the importance of self-cultivation as an important initiator of compassionate behaviors, enabling organizational and team development. In their view, self-cultivation and compassion are closely intertwined and prerequisites for organizational growth, even though the relationship may not be straightforward. Karakas and Sarigollu (2013) espouse the role of benevolent leadership in fostering a compassionate organization through the four elements of: spiritual depth, ethical sensitivity, positive engagement, and community responsiveness. Table 6 presented below reflects the definitional views of the different authors.

**Table 6:** Definitional views of different authors

S.No	Author	How compassionate leadership is defined
1	Banker and Bhal (2018)	Associates compassionate leadership to a mindset
2	Vogus et al. (2020)	Associates compassionate leadership to servant leadership
3	Hewison et al. (2019)	Associates compassionate leadership to servant and resonant leadership
4	Oruh et al. (2021)	Associates compassionate managerial leadership to an act of love, care and selflessness
5	Hougaard et al. (2020)	Associates wisdom combined with compassion to wise compassionate leadership
6	Shuck et al. (2019)	Associates integrity, empathy, accountability, authenticity and helping to compassionate leadership
7	Willis and Anstey (2019)	Associates qualities of attending; understanding; empathetic and helping to compassionate leadership
8	de Zulueta (2016)	Associates compassionate leadership to servant leadership
9	Karakas and Sarigollu (2013)	Associates compassionate leadership to benevolent leadership

A review of the selected literature establishes the point that the definition of compassionate leadership is ambiguous, fragmented, blurs and resonates with other leadership constructs and approaches, such as servant leadership, resonant leadership, benevolent leadership, transformational leadership or ethical leadership. Shuck et al. (2019) acknowledge the connection compassionate leader behaviours have to other leadership typologies, yet at the same time dismiss the notion that these typologies are the same. This is reminiscent of the many dimensions into which leadership' itself has been cast with many over-lapping meanings adding to the confusion. Nevertheless, there are sufficient pointers to permit a rough sketch of a classification of the construct compassionate leadership.

Based on the literature and definition synthesis, we propose “compassionate leadership can be conceived as a personality encompassing the traits and behaviours of compassionate love, care, selflessness, wisdom, integrity, empathy, accountability, authenticity, presence, dignity, self-compassion and self-cultivation as a matter of inducing, motivating, influencing, persuading people to achieve their personal and organizations growth”. However, we concede that this is a broad and general definition which required further development.

### Existing measures for compassionate leadership (RQ2)

A review of the articles reveals that the construct of compassionate leadership is still in an embryonic stage. Robust measures to capture compassionate leadership are essential for the establishment of a common conceptual base, especially when the existing body of knowledge on compassionate leadership is limited and scattered across the literature. Unfortunately, we have only come across one study which has attempted to develop the measures of compassionate leadership. Shuck et al. (2019) concede the lack of previously developed items and produce their own items on six different themes derived from the literature: Empathy; Presence; Integrity; Authenticity; Dignity and Accountability. More work is required to test the generalizability and transferability of the six themes of compassionate leadership in different contexts. Other studies such as Balasubramanian and Fernandes (2022) have used compassion as sub-construct to measure crisis leadership. The four key measures for this construct include 'active listener to employee concerns', empathy, employee needs are central to decision making, and encourage employee to openly and honestly express their concerns. This four-item construct had strong convergent and discriminant validity and had a reliability of 0.92 (Cronbach's alpha).

Still, there is a great scope for development of a robust measure imbibing other dimensions of leadership as discussed in the previous sections to measure compassionate leadership behaviour. Lack of reliable quantitative measures had stalled researchers' efforts in evaluating compassionate leadership.

Not only should compassionate leadership be measured from the 'givers' (leaders) perspective but also from the 'receivers’ side (follower perspective). Due to lack of studies involving the measures, one is unable to contrast and compare the different measures employed by different studies. However, as the field of study develops further, we expect that more studies would emerge with robust measures.

### Proposed dimensions of compassionate leadership (RQ3)

It was evident from our review, compassionate leadership is a complex and multifaceted phenomenon, encompassing several dimensions. The six key dimensions of compassionate leadership derived from the literature are as follows:

#### Empathy

Empathy is defined as one’s ability and propensity to sense the feelings of people in emotional distress and to re-experience these feelings oneself (Salovey et al. 2003). This includes cognitive, emotional, and behavioural tendencies to be attentive to others’ feelings and to understand the world from their point of view. Further, empathy at the workplace includes the need to listen attentively, relate to and understand others, as well as clarify others' perspectives. For example, feeling the pain of someone for a particular issue and taking action to alleviate that pain (Vogus et al. 2020). The concept of empathy in the leadership context is understanding employees’ perspectives, thoughts and/or feelings and taking action (Shuck et al. 2019). Empathy toward employees is considered a critical component of compassionate leadership. Empathetic leaders show feelings of warmth and concern for employees in distress. Several studies highlight empathy as the foundation for building compassionate leadership. According to Banker and Bhal (2018), leaders should empathize with people’s issues and look forward to building a compassionate organization in order to yield positive organizational benefits. Empathetic concern towards employees can successfully build compassionate culture by ingraining ethical/moral virtues in people, creating a conducive workplace, and building trust in people for the organization. For instance, when helping an employee through a challenging task, a leader may notice the struggle, have empathy toward the employee and ask how they can help.

#### Openness and communication

Creating a culture of openness is important for promoting compassion at workplace. Hence, compassionate leaders promote openness at workplace. Communications that are honest and transparent, being factual, frequent, iterative, clear and direct are critical aspect of compassionate leadership. Employees respond so much better to the known (even if the news isn’t great) than the unknown (which tends to fuel more anxiety) or, even worse, misleading half-truths or irresponsible optimism (Balasubramanian and Fernandes 2022). Constant and open communications from leaders was found to helped alleviate the negative feelings developed at workplace. The importance of open and transparent communication was evident during the COVID-19 pandemic (Balasubramanian and Fernandes 2022).

#### Physical, mental health and well-being

Compassionate leaders are expected to take actions to protect, maintain, or restore wellbeing. physical, mental health and wellbeing of employees especially for stressful occupational conditions. This include balancing employee workload, increase safety precautions at workplace, promote mental health among others. Leaders that advance and improve working conditions in terms of physical, mental and social wellbeing, are more likely to have employees perceiving their leaders’ management style as positive (i.e., manages who are supportive, consider their concerns, empower them, and listen, including co-operating with them and providing counselling) (Mayer and Oosthuizen 2020). This include providing psychological support and coping mechanisms. These employees demonstrate high levels of psychological connection, engagement and performance.

#### Inclusiveness

Leader inclusiveness or “words and deeds exhibited by leaders that invite and appreciate others’ contributions” is an important component of compassionate leadership (Nembhard and Edmondson 2006). Inclusiveness involves looking after employees at all levels, and functions including outsourced employees. Compassionate leaders promote inclusive work culture, involve and engage in inclusive decision-making wherein the leader will take the employees in confidence and seek their feedback/advice irrespective of their designation. Previous studies have shown that inclusiveness towards employees are associated with higher level of psychological safety, and diminishes the inhibiting effects of status differences and was associated with greater engagement in quality improvement work. Inclusiveness increases feelings of equality via reduced status distance, and therefore foster compassion among employees and between employees and customers (Vogus et al. 2020).

#### Integrity

The theme of integrity revolves around a sense of professional transparency, ethical behavior, fairness, trust, credibility and a personal and professional alignment with what a leader would say and do. Integrity is a core component of compassionate leadership. It helps build a level of employee credibility and trust on their leaders (Shuck et al. 2019). Similarly, compassionate leaders are expected to be fair with employees. Such leaders keep their word or promises, even when they were being pulled in different directions with competing priorities.

#### Respect and dignity

Compassion underpins respectful relationships. Dignity involves acknowledging the value and contribution of each person. For a compassionate leader, every employee (even if they are struggling) is of value to the organization so they treat them with interest and respect. By treating people with respect, valuing employees' contributions, and honoring them, employees will develop a great sense of dignity at work (Shuck et al. 2019).

### Leader, follower and organizational benefits of compassionate leadership (RQ4)

It was evident from the review that compassionate leaders have stronger and more-engaged followers. Compassionate leadership helps leaders to focus on the positive, look beyond cultural differences and see the good in others. It helps leaders to be appreciative and open-minded and minimizes cultural and religious stereotypes. The compassionate leader is well placed to recognize and nurture every member of the team's strengths and talents and develop their skills and confidence (Willis and Anstey 2019).

Frost (2003) examines the actions of leaders and organizations in creating emotional pain and points out how the emotional pain, if unacknowledged and unaddressed, leads to toxicity in the workplace affecting performance. The study argues that leaders must recognize this situation and take steps to alleviate the suffering if their organization has to remain productive. He asserts that emotional pain can be turned from an obstacle to a positive force for change if it is acknowledged and appropriately managed. In achieving this, compassionate leadership can ensure that the value of staff wellbeing is recognized and, more importantly, that staff should feel that they are being cared for and not ‘just treated’ (Willis and Anstey 2019). Foster (2021) opines that a leader must understand the extraordinary pressure and the circumstances employees work under and encourage kindness, find time to listen to people's frustrations, show empathy, and be aware. Karakas and Sarigollu (2013) echo a similar opinion when they assert that benevolent leaders, i.e., who create observable benefits, actions, or results for the common good, thus contributing to the creation of compassionate organizations. Oruh et al. (2021) reported that compassionate leadership can help employees respond to their concerns of “fear of job (in)security”, “healthcare risks” “work overload, underpayment, and delayed payment” being the main causes of stress for employees during COVID-19.

From an organizational perspective, compassionate leadership yields many favorable benefits to the organization; for example, compassionate behavior with employees during turbulent times leads to increased employee commitment and  reduced absenteeism (Dutton et al. 2006). Firms with more-compassionate leaders have better collaboration, lower employee turnover, and followers who are more trusting, more connected, and more committed to the organization (Hougaard et al. 2018). Compassionate leadership creates the necessary conditions for innovation among individuals, teams, and organizational levels (Hewison et al. 2019). Shuck et al. (2019) found compassionate leadership to have a negative influence on employee turnover and a positive correlation between compassionate leadership, employee engagement, and employee psychological wellbeing. Compassionate leadership positively impacts employees, encouraging creativity in problem-solving and a high level of engagement (Mayer and Oosthuizen 2020).

### The future of compassionate leadership studies (RQ5)

Future research directions are drawn from the limitations of existing studies. One of the major limitations of this study is the paucity of studies focused on compassionate leadership in the field of management. This paucity while posing a limitation also provides a good scope for further research avenues. In this section, we offer our views on future course of studies on compassionate leadership.

Firstly, the concept of compassionate leadership has been widely discussed in industry publications and sparingly in the academic journals. While there appears to be general understanding of what compassionate leadership means, there is by no means any agreement on what exactly is compassionate leadership, its boundaries or its conceptualization. The systematic literature review reveals that studies never specifically define what they mean by compassionate leadership and simply use the term and assume the reader understands the concept. While we have attempted to provide a broad and general definition in line with Bass (1990, p.19), this requires further development. The concept of compassion transcends both time and discipline and has been viewed by different scholars through different lenses depending upon their theoretical tradition and empirical conversation from where they approach the topic (Frost et al. 2006) and so is the concept of leadership. With both these concepts now intertwined and dimensions of both compassion and leadership transcending several disciplines, it requires a rigorous examination for an acceptable definition to be proffered. As pointed out by Bass (1990) a definition should do more than identify leaders and indicate the means by which they acquire or dispense their positions and should account for the maintenance and continuation. Defining compassionate leadership can be a long-drawn-out but a meaningful and worthy discussion for scholars to turn their attention upon.

Secondly, while compassionate leadership is being mooted as a panacea for troubled times and as an effective leadership approach, studies should also focus on the down-side or disadvantages to the approach, particularly in light of the fact that capitalism as an economic model can bring about tension and contradictions in the work environment and therefore leave little or no room for compassion. More particularly when practicing compassion, it may be construed as being’soft’ causing the receiver to take advantage of the giver (Poorkavoos 2016).

Thirdly, the concept of compassion is often rarely expected and experienced in organization climates; this is particularly very visible in the public sector. How then can a leadership approach of this nature be applicable and prove to be valuable in the private sector? For example, one may expect this quality to be a most valuable asset for policy makers in the public sector when interacting with the local citizenry and their constituents, but will it serve in the long run as an approach to general leadership in the private sector?

Fourthly, there is a great scope for development of robust measures imbibing other dimensions of leadership to measure compassionate leadership behavior. Previous literature has largely failed to empirically examine compassionate leadership as a multi-dimensional construct. Future studies could focus on developing a measurement model incorporating the various sub-dimensions of compassionate leadership such as empathy, care, integrity etc.

Fifthly, not only should compassionate leadership be measured from the leaders’ perspective but also from the followers’ perspective. Measuring compassionate leadership from both leader and follower perspective is critical given that discrepancies are often found between leaders’ self-perception and followers’ perception of leadership (Sehgal et al. 2021). A greater discrepancy in the leader–follower perception could lead to surprise, consternation, disbelief, or emotional distress among employees, which could lead to a poor organizational culture and employee productivity. Such understanding is critical for narrowing the leader–follower perception gap and achieving congruence.

Sixthly, it is clear from the review that hardly any studies have examined compassionate leadership in the context of the Fourth Industrial Revolution (Industry 4.0). Industry 4.0 is synonymous with an environment where technology and human interaction are hand-in-glove and enmeshed. The resultant outcome is expected to produce desired results and successful outcomes of strategic organizational initiatives. Smart systems demand high levels of innovation and creativity, digitalization, and artificial intelligence, which may contribute to high levels of stress experienced at the workplace. Technology is employed for generating efficiency and benefits for the organization, but the same technology can also induce stress and emotions such as frustration, disorientation, sadness, insecurity, and ambiguity, among many other negative emotions among the employees. It is unclear how compassionate leadership could create and provide the appropriate guidance during the industry 4.0 to address the employee challenges that can negatively impact organizational goals.

Seventhly, most of the studies on compassion and compassionate leadership emanate from the medical and healthcare field (de Zulueta 2016; Hewison et al. 2018, 2019; Hofmeyer et al. 2020; Jones et al. 2021; Landers et al. 2020; Papadopoulos et al. 2021; Willis and Anstey 2019), while the field of management suffers from a deficit leaving the field wide open for a range of studies.

Finally, it is essential to look at compassionate leadership from a critical standpoint, given the realistic situation of most organizations. Given the increasingly competitive landscape, it is very likely that the compassionate leader is seen as someone who is "weak" or "being soft" or trying to please people by giving them what they want; rather than giving people what they need, such as tough feedback.

We summarize the eight recommendations for future research in Table 7 below.

**Table 7:** Recommendations for future research. Research areas and questions

S.No	Topic	Research area/questions
1	Definitional ambiguities	While research has offered different views on compassionate leadership, the fundamental question, what is compassionate leadership, still remains. An acceptable common definition of compassionate leadership is needed
2	Disadvantages of compassionate leadership approach	While compassionate leadership is being mooted as a panacea for troubled times, studies should also focus on the down-side or disadvantages for a balanced view
3	Compassionate leadership in the private sector domain	How can compassionate leadership serve in the long run as an approach to general leadership in the private sector?
4	Robust operationalisation	A robust operationalization of compassionate leadership in organizations will help ensure the concept is understood and measured consistently in different fields. Studies could focus on developing a robust measurement model incorporating the various sub-dimensions of compassionate leadership such as empathy, integrity, opennes and communication, respect and dignity, physical, mental health and well-being, inclusiveness
5	Views and measures from both leader and follower perspectives	How is compassionate leadership viewed through a combined lens of both leader and follower? What is the gap in the perception of both leader and follower and how the gap could be narrowed for achieving congruence. An empirical study would be useful
6	Compassionate leadership in the context of Fourth Industrial Revolution (Industry 4.0)	Technology is a double-edged sword. While it generates efficiency and benefits, it also induces negative tendencies among users. How can compassionate leadership create and provide appropriate guidance in the context of Fourth Industrial Revolution to address employee challenges that can negatively impact organizational goals?
7	Deficit of studies in the field of management	Compassion and compassionate leadership studies prevail mostly in the medical and healthcare field. The field of management is a fertile ground wide open for a range of empirical studies
8	A’soft’ or a’weak’ approach	How can leaders adopt a’compassionate’ approach without being perceived as being soft or weak?

### Study limitations

Although this study was rigorously done, we acknowledge the limitations, especially those concerning the literature sampling criteria and analysis. The final list of studies considered may not be exhaustive because of the limitations in the keywords searched and the databases used. Regarding the findings, it is important to note that the proposed six dimensions of compassionate leadership are not intended to be the final word on the subject. Rather, they represent an initial exploration into the dimensions of compassionate leadership. There is a need for further research to refine, validate and extend these dimensions and develop measurement items to fully capture each aspect. Additionally, it's essential to consider that compassionate leadership does not exist in isolation, but is rather interwoven with other leadership styles, such as hybrid leadership, benevolent leadership and servant leadership. It would be interesting to explore the similarities and differences between compassionate leadership and other leadership styles in future studies. Ultimately, our study represents a first step in understanding the complexities of compassionate leadership, but there is still much more to uncover and explore in this area.

However, despite these limitations, the contributions of this study are novel and of significant relevance for both practitioners in advancing our understanding of compassionate leadership. For researchers, this study provides a solid theoretical foundation that may greatly facilitate further empirical work in this domain.

## Implications and conclusions

This study explored more than two decades of studies on compassionate leadership. This study advances knowledge and points out the gaps in the previous literature on compassionate leadership. For instance, this study has identified several trends, consensus, conflicts, approaches, and methods to better understand and improve the concept of compassionate leadership and guide future research. The implications of this study are manifold, as discussed below:

### Practical implications

The review of the articles indicates that compassionate leadership has the potential to have positive effects on both the leadership and the workforce. Training programs can be developed around the different dimensions of compassionate leadership by developing a deeper understanding of what it means to be a compassionate leader. The key practical implications of the review articles are summarized in Table 8.

**Table 8:** Key practical implications of the review articles

S.No	Author/s	Title	Journal	Practical implications
1	Foster (2021)	Finding the right leadership style	British Journal of Nursing	Compassionate leadership activities have many positive outcomes, at all levels of the health sector, from individuals and teams. Compassionate leadership is a powerful facilitator at each stage of the problem-solving process Showing empathy and understanding is crucial when staff need to know more than ever that their leaders care about them
2	Papadopoulos et al. (2021)	The Importance of Being a Compassionate Leader: The Views of Nursing and Midwifery Managers from Around the World	Journal of Transcultural Nursing	Transcultural models and practices of ethical leadership should be developed to establish nurturing health care working environments from which all stakeholders can benefit An effort to “institutionalize compassion” and cultural competence should be adopted throughout the organization with collective holistic learning strategies and high levels of staff support and engagement Providing training in coping and self-compassion strategies is imperative that will encourage nursing and midwifery managers to show compassion and to nurture the values that underpin professional codes of practice
3	Jones et al. (2021)	Social work leadership in a medical school: A coordinated, compassionate COVID-19 response, Social Work in Health Care	Social Work in Health Care	Medical schools should consider including social work leadership in their structures as social workers can quickly adapt and organize efforts to integrate psychosocial and health care with a focus on equity and justice
4	Oruh et al. (2021)	Exploring compassionate managerial leadership style in reducing employee stress level during COVID-19 crisis: the case of Nigeria	Employee Relations	During extreme situations such as a pandemic, drastic and managerialist-driven work measures could trigger high stress levels, low productivity and absenteeism among employees. Organizations can benefit by implementing compassion-driven policies which are inclusive and responsive to the stressors facing employees
5	Vogus et al. (2020)	Creating a compassion system to achieve efficiency and quality in health care delivery	Journal of Service Management	Proposes a model of compassion system which fosters a compassion climate focusing on how to efficiently reduce suffering and provide high quality care and care experiences in health care organizations
6	König et al. (2020)	A blessing and a curse: How CEOs' trait empathy affects their management of organizational crises	Academy of Management Review	Presents a conceptual model on how and why empathy is crucial for CEOs to succeed at their job, while also explaining the potential downsides of higher levels of CEO empathy. Both high and low levels of empathy are likely to harm companies’ abilities to resolve organizational crises
7	Hofmeyer et al. (2020)	Fostering compassion and reducing burnout: How can health system leaders respond in the Covid-19 pandemic and beyond?	Nurse Education Today	Implement relevant organizational interventions to reduce caregiver burnout and promote engagement and compassionate practice during times of pandemic and beyond
8	Mayer and Oosthuizen (2020)	Sense of coherence, compassionate love and coping in international leaders during the transition into the fourth industrial revolution	International Journal of Environmental Research and Public Health	Sense of coherence (SOC) interventions in organizations need to be implemented which would foster positive feelings, thereby promoting affection and interest in others, increasing emotive encounters and meaning. Compassionate Love (CL) needs to be actively implemented into leadership, is connected with SOC and is a social and intra-personal coping mechanism for leaders transitioning into the fourth industrial revolution
9	Hougaard et al. (2020)	Compassion leadership is necessary-but not sufficient	Harvard Business Review	Proposes to develop more self-compassion; putting oneself in others shoes; adopt a daily compassion practice; practice candid transparency; be candid in delivering guidance even if it’s difficult to hear
10	Landers et al. (2020)	Nurses’ and midwives’ views of the “Leaders for Compassionate Care Program”: A qualitative analysis	Collegian	Initiatives to sustain and clarify the meaning of the concept of compassion should be an ongoing aspect of professional education for qualified nurses and midwives Embedding and maintaining compassionate care requires careful planning and resources to equip practitioners to be leaders in the delivery of compassionate care
11	Hewison et al. (2019)	Compassionate leadership in palliative and end-of-life care: a focus group study	Leadership in Health Services	Leadership development needs to be deeply embedded in and informed by the context and the challenges that leaders in the organization face collectively in the specific organization context and requires conversations and learning with people who share that context Context-specific action learning may be a means of further developing compassionate leadership capability in palliative and end-of-life care and more widely in healthcare settings
12	Shuck et al. (2019)	Does Compassion Matter in Leadership? A Two‐Stage Sequential Equal Status Mixed Method Exploratory Study of Compassionate Leader Behaviour and Connections to Performance in Human Resource Development	Human Resource Development Quarterly	Compassion offers a way forward for thinking about how work is getting done, not just how much (Frost 2003) Training programs can be developed that focus on how to listen with empathy and presence, how to be attuned to cues that signal employees are dealing with an emotional issue, how to reframe organizational messages that could cause resistance and trauma, as well as how to build social and political capital to protect teams from internal or external challenges
13	Lown et al. (2019)	Can Organizational Leaders Sustain Compassionate, Patient-Centred Care and Mitigate Burnout?	Journal of Healthcare Management	Leaders must define the organizational conditions and implement processes that support professionals' innate compassion and contribute to their well-being rather than address burnout later through remedial strategies
14	Tzortzaki (2019)	The Need for Teaching Compassionate Self-Leadership in a University Setting	12th Annual Conference of the EuroMed Academy of Business	This study makes a strong argument for the instrumental role of self-compassion in sustaining a leader’s compassionate, servant approach in the long-term and for the need of more compassionate leaders in today’s materialistic cultures Self-leadership training courses can positively affect the ability of an individual to become a self-leader
15	Willis and Anstey (2019)	Compassionate leadership in district nursing: A case study of a complex wound	British Journal of Community Nursing	The framework developed to illustrate compassionate leadership in action can be put into practice to meet the needs of team and nurses delivering person-centered care
16	Hewison et al. (2018)	Leading with compassion in health care organisations: The development of a compassion recognition scheme-evaluation and analysis	Journal of Health Organization and Management	The model of compassion can be used to demonstrate what compassion “looks like”, and what is expected of staff to work compassionately
17	Hougaard et al. (2018)	Assessment: Are You a Compassionate Leader	Harvard Business Review	Proposes an assessment to help understand how wisely compassionate one is along with tips on how to become a compassionate leader
18	Banker and Bhal (2018)	Understanding Compassion from Practicing Managers’ Perspective: Vicious and Virtuous Forces in Business Organizations	Global Business Review	Highlighted the role of empathetic leadership as a foundation to build compassionate organization. Competent empathetic leadership can successfully build compassionate culture by ingraining ethical/moral virtues in people, creating conducive work place and building trust in people for the organization Considering the myriad challenges faced by organizations, organizations may be addressed having, ‘an absence of compassion’ rather than being called as ‘non-compassion’
19	MacArthur et al. (2017)	Embedding compassionate care in local NHS practice: Developing a conceptual model through realistic evaluation	Journal of Research in Nursing	Embedding and sustaining compassionate care demands strategic vision and investment in a local infrastructure that supports relationship- centered care, practice development and effective leadership at all levels
20	Milner (2017)	Rekindling the flame of community through compassion – a call for leadership toward compassionate community	Aotearoa New Zealand Social Work	Not stated explicitly. However, the paper argues that Relational community development practice driven by compassion enhances social trust, fairness and justice. It is sustained by creativity and hope, and by workers who have a sense of spirituality’s power to improve wellbeing
21	de Zulueta (2016)	Developing compassionate leadership in health care: an integrative review	Journal of Healthcare Leadership	Education and training in leadership skills also needs to be collective, i.e., for all those working in health care organizations, including those who do not consider themselves as leaders, in order to create a collective leadership culture of compassion and collaboration Training in the form ‘multi-disciplinary “in-house” team coaching and training Managers will need to be trained in coaching skills to support staff Develop programs that promote shared, “post heroic”, collaborative, distributed leadership, beginning with the issues patients face and learning in the context of application Need to nurture and cultivate facilitators, team builders, mentors, and coaches in large number who are creative, transformative, and adaptive Need for individualized senior leadership development, with a focus on servant and adaptive leadership attributes and practices Leadership needs to embody and model compassionate attitudes and behaviors and develop self-compassion and emotional resilience
22	Wei et al. (2016)	Top executive leaders’ compassionate actions: An integrative framework of compassion incorporating a Confucian perspective	Asia Pacific Journal of Management	Self-cultivation and compassion are closely intertwined. Therefore, leaders can gain by aligning self-cultivation and compassion. Leaders can also ascertain how to balance self- and other interests. This integration of Self-cultivation and compassion can be powerful in bringing people and organizations together
23	Dewar and Cook (2014)	Developing compassion through a relationship centred appreciative leadership programme	Nurse Education Today	Full text unavailable
24	Seco and Lopes (2014)	Between compassionateness and assertiveness: A trust matrix for leaders	Journal of Industrial Engineering and Management	Compassionate behaviors tempered or complemented with assertive behaviors could be effective for leadership in organizations
25	Karakas and Sarigollu (2013)	The Role of Leadership in Creating Virtuous and Compassionate Organizations: Narratives of Benevolent Leadership in an Anatolian Tiger	Journal of Business Ethics	Using the benevolent framework, organizations can enable a positive community where employees feel authentic and connected with their inner selves, their co-workers, and their community Flatter organizations where leadership is distributed or shared can benefit with benevolent leadership Leadership programs which foster benevolent leadership perspective and best practices of benevolent leaders could be organized by organizations Organizations can support by encouraging, reinforcing, and rewarding benevolent leadership behaviors Organizations could recruit individuals who possess benevolent leadership characteristics to create a benevolence-oriented organizational culture The results of this study can be used by leaders to develop self-awareness through reflection Using benevolent leadership framework, leaders can create supportive team and work environments Benevolent leadership model has the potential to create virtuous and compassionate organizations
26	McKimm and O’Sullivan (2013)	Personality, self-development and the compassionate leader	British Journal of Hospital Medicine	Clinical leadership and doctors’ engagement in management is vital to ensuring the focus on providing compassionate care to all care receivers through behaviors expected by the patients and public Assessment of behaviors based on demonstration of personal qualities should be embedded in multisource feedback, appraisals and performance reviews as well as in routine workplace-based assessment for trainees and students
27	Hornett (2012)	Compassionate leadership	British Journal of Nursing	Need to integrate compassionate care into the work culture without additional facilitation
28	Rynes et al. (2012)	Care and Compassion Through an Organizational Lens: Opening Up New Possibilities	Academy of Management Review	The review discusses several practical implications derived from the articles published in the special topic forum
29	Dutton and Workman (2011)	Commentary on 'Why Compassion counts!': Compassion as a Generative Force	Journal of Management Inquiry	A focus on compassion in organizations lights up suffering that is often hidden and also reveals the healing -force of human-to-human compassion Adopting a compassion lens enables new ways to consider individuals, groups and collective capabilities to be acknowledged and valued
30	Martinek et al. (2007)	The development of compassionate and caring leadership among adolescents	Physical Education & Sport Pedagogy	Leadership skills can be fostered in adolescents by teaching sport and life skills
31	Boyatzis et al. (2006)	Developing Sustainable Leaders Through Coaching and Compassion	Academy of Management Learning & Education	Leaders are more likely to be effective if they are physiologically and psychologically balanced Coaching with compassion may provide the platform for sustainable leadership effectiveness and an effective approach for developing leaders The integration of coaching, compassion, and leadership would result in a steady stream of capable leaders for the organization Coaching, beyond the traditional notion of advising, could become a crucial pedagogical method
32	Dutton et al. (2006)	Explaining compassion organizing	Administrative Science Quarterly	The Theory of Compassion Organizing suggests five core mechanisms central to explaining the pattern of compassion organizing. (1) Attention to pain triggers (2) Generation and spread of emotions (3) Trust and legitimacy are resources critical to compassion organizing (4) Knowledge, experience, emotional and practical intelligence could contribute to compassion organizing (5) Symbols could enrich organizational processes towards contributing compassion organizing
33	Kanov et al. (2004)	Compassion in Organizational Life	American Behavioural Scientist	Important processes of propagation, legitimation and coordination could enable the transformation of compassion into a collective phenomenon
34	Frost (1999)	Why compassion counts!	Journal of Management Inquiry	Invokes the importance of compassion in organizational life
35	Brouns et al. (2020)	Leadership beyond narcissism: On the role of compassionate love as individual antecedent of servant leadership	Administrative Sciences	Individuals who display compassionate love have the potential to be good servant leaders who promote sustainability of the organizations Organizations should consider ways and means to make servant leadership ideas accessible to leaders
36	Paakkanen et al. (2021)	Awakening Compassion in Managers- a New Emotional Skills Intervention to Improve Managerial Compassion	Journal of Business and Psychology	Leaders and organizations to invest in building compassionate cultures through compassion training, decreasing the levels of obstacles to compassion or strengthening the sub-process of compassion process
37	Basran et al. (2019)	Styles of Leadership, Fears of Compassion, and Competing to Avoid Inferiority	Frontiers in Psychology	Leadership training for individuals who adopt prosocial and antisocial leadership styles will need different orientations in training
38	Zoghbi-Manrique-de-Lara and Viera-Armas (2019)	Does Ethical Leadership Motivate Followers to Participate in Delivering Compassion?	Journal of Business Ethics	A preventive compassion-based strategy is advisable to cultivate or strengthen healthy employee peer–peer feelings to recognize instances of need or pain Managers must design actions that make leaders able to communicate value and support for staff’s efforts to bring peers from moral exclusion and physical isolation Develop education and training programs targeted at reducing stereotyping, increasing cultural sensitivity, and developing the skills needed to work in multicultural environments
39	Eldor (2018)	Public Service Sector: The Compassionate Workplace-The Effect of Compassion and Stress on Employee Engagement, Burnout, and Performance	Journal of Public Administration Research & Theory	Positioning compassion as a contributor to a high-quality, service oriented public system may increase civilian trust and public service agencies Receiving compassion may also positively contribute to public service employees to their stressful environment caused by demanding public service interactions and administrative workload conditions The receipt of compassion from supervisors has a strong positive impact upon the employee work performance behaviors The actions of public service managers should be driven by rationality, but executed with sensitivity
40	Coffey et al. (2019)	The impact of compassionate care education on nurses: A mixed-method systematic review	Journal of Advanced Nursing	A strong need to establish novel education programs that not only promote compassionate care delivery at all levels of care, but also promote self‐compassion in nursing
41	Lilius et al. (2008)	The contours and consequences of compassion at work	Journal of Organizational Behaviour	Those experiencing compassion in an organizational setting feel distinct positive emotions, such as pride, gratitude, inspiration, and ease. It is also a simple but powerful way of strengthening emotional well-being at work leading to a broadening of people’s mindset, and building their intellectual, physical, social and psychological resources

The synthesis of practical implications in these studies reemphasizes the fact that empathy, understanding, and self-compassion are critical elements in creating a positive work environment and achieving better outcomes, success, and well-being of individuals, teams, and organizations across various sectors such as healthcare, public service, and business(Foster 2021; Hougaard et al. 2020; König et al. 2020). The positive outcomes include increased trust, collaboration, and engagement among team members. Incorporating compassion into leadership styles, training programs, and organizational cultures is emphasized to reduce burnout, promote engagement, and improve overall well-being (Hewison et al. 2019; Hofmeyer et al. 2020; Jones et al. 2021; Landers et al. 2020; Oruh et al. 2021; Papadopoulos et al. 2021; Shuck et al. 2019) Additionally, the role of benevolent leadership, coaching with compassion, and integrating coaching, compassion, and leadership is discussed as crucial for developing capable leaders(Boyatzis et al. 2006; de Zulueta 2016; Karakas and Sarigollu 2013).

The common thread across these implications is the recognition that leadership is not just about getting things done but also about how things are done (Shuck et al. 2019). Leaders who practice compassion can create supportive, collaborative, and inclusive work environments where people feel valued, connected, and engaged.

Moreover, the implications provided in these studies in Table 8 also highlight the need for education and training in leadership skills, particularly in compassionate leadership. This includes training in empathy, self-compassion, coaching, and emotional resilience(Banker and Bhal 2018; de Zulueta 2016; Hougaard et al. 2020; König et al. 2020; Shuck et al. 2019; Tzortzaki 2019). In addition, these studies emphasize the importance of creating a collective leadership culture of compassion and collaboration, where all those working in an organization, including those who do not consider themselves as leaders, are equipped with leadership skills and contribute to a culture of compassion (de Zulueta 2016).

The analysis also indicates that compassionate leadership is not limited to healthcare settings but is relevant in various organizational contexts. This includes public service agencies, business organizations, and educational institutions. These studies suggest that leaders must define the organizational conditions and implement processes that support professionals' innate compassion and contribute to their well-being rather than address burnout later through remedial strategies (Lown et al. 2019).

Overall, the commonalities in these implications underscore the importance of compassionate leadership and the need for education and training in leadership skills. The results emphasize that leaders must create supportive work environments that foster collaboration, inclusivity, and compassion and to that end, practical interventions can be tailored to different contexts. For instance, in the case of healthcare, specific interventions that can be implemented include providing psychological support for officers suffering from compassion fatigue or establishing assessment programs to identify and prevent burnout (Hewison et al. 2019). Other studies suggest that leadership training should be collective and that leadership development should be informed by the context and challenges faced by leaders in specific organizations. There are also suggestions for integrating compassion into the work culture without additional facilitation and creating a supportive team and work environment through coaching with compassion (Hornett 2012).

The Fourth Industrial Revolution and its attributes have already been mentioned. Its effects on business organizations are profound and have caused many organizations to revamp their leadership direction in order that they continue and maintain their profit focus and maintain their competitive positions. Banker and Bhal (2018) remind us that organization prima facie is a profit-making entity than a social entity. Developing an organization as compassionate or non-compassionate requires the critical assessment of: (a) the intent with which it operates, (b) the kind of environment it operates within, and (c) the availability of monetary or non-monetary resources it has for different organizational and social activities. Banker and Bhal (2018) are of the opinion that in spite of having any of the three elements “missing” in an organization, the motivation for compassion can be infused by the empathetic leadership practices. Thus, it is up to the leadership to instil these practices as they are determined to have an impact on the profit-making motives of the organization. Organizations may thus continue to analyse their leadership approaches which may include the compassionate leadership from this angle.

We have long passed the era when it was believed that leaders were born into a heritage or succession line of leaders. Today, we are taught that we can all become leaders if inspired to do so and that the potential to be successful can be attained through education and training while developing the tools to motivate and convince others to accept and follow our beliefs. In addition, the experience will allow us to gain the fortitude to enhance our leadership skills, hinting that it is a continuous process as new and different challenges are confronted in the process. However, the basic traditional leadership qualities must exist in order to serve as a solid foundation from which one might build a career as a leader. These include, having and maintaining strong moral values; having a vision that provides fuel for striving to reach desired goals; and to be forward thinking, which the leader’s followers will expect of him/her.

What then has changed that would allow the compassionate leadership approach to be a viable and formidable approach when adopted by a leader to guarantee him/her success in achieving the vision? In the current contemporary business environment, a variety of factors have changed which may necessitate the adoption of the compassion leadership approach and prove to be beneficial for leaders to attain their objectives and goals. The following factors are contributing to major changes in the organizational environment that are challenging the leaders: Global work relationships; Developing transcultural environment with balanced relationships at the workplace; Technology and Digitalization—both are rapidly and constantly evolving; Economic downturn and finally, the global pandemic and its devastating impact.

A significant aspect of the global relationship factor is the migration of workers, which is a major contributively factor in organizations becoming the transcultural environments where it has become necessary for leadership and management to balance relationships to produce positive outcomes and attain strategic objectives. Woven into the fabric of the transcultural environment is the thread of technology in the form of digitalization and/or artificial intelligence, which brings into play the complexities and dynamism of the drama that unfolds in contemporary business organizations. Interwoven in this scenario is the calamitous event of the epidemic, which has left a trail of devastation in its wake. Compassionate leadership may well be the most beneficial and appropriate approach that organizational leaders may adopt to ride the tidal wave of the current climate in which they find themselves in.

### Theoretical and research implications

There is little doubt that the concept of compassionate leadership is growing in importance, as is evident with the gradually increasing attention of academia and business alike. Some of the key theoretical and research implications are as follows:

- A comprehensive literature review-based assessment of compassionate leadership has not been previously undertaken and constitutes the novelty of this work.
- The study highlights several research gaps in the literature to enhance our understanding of compassionate leadership.
- Six dimensions of compassionate leadership are proposed.
- A clear future research agenda is provided.
- This study has managed to bring some degree of consensus to the different definitions of compassionate leadership by providing a ‘new definition’ of compassionate leadership.

However, more research is required on compassionate leadership, especially in developing validated constructs and measures to appreciate the application of compassionate leadership in the organizational literature and also for it to take its rightful place as one of the accepted leadership styles.

## Data Availability

The author confirms that all data generated or analysed during this study are included in this published article.
